# The mediating role of fear of childbirth in the relationship between intolerance of uncertainty and cesarean section preference among pregnant women

**DOI:** 10.3389/fpsyt.2025.1700807

**Published:** 2026-01-12

**Authors:** Feng Zhang, Yuqing Zan, Min Liu, Shanshan Hu, Ningying Zhou, Wenjing Zhang, Na Wang

**Affiliations:** 1 Wuxi School of Medicine, Jiangnan University, Wuxi, Jiangsu, China; 2 Department of Nursing, Wuxi Maternal and Child Health Hospital, Wuxi School of Medicine, Jiangnan University, Wuxi, Jiangsu, China

**Keywords:** antepartum, cesarean section, fear of childbirth, intolerance of uncertainty, maternal mental health, mediating effect

## Abstract

**Objective:**

This study aims to investigate the relationship between intolerance of uncertainty and preference for cesarean section, and to explore whether fear of childbirth mediates the relationship between intolerance of uncertainty and preference for cesarean section.

**Methods:**

A total of 310 pregnant women in the third trimester who attended outpatient clinics at Wuxi Maternal and Child Health Hospital from July to September 2024 were included in this study. The survey instruments used included a Sociodemographic Information Questionnaire, Intolerance of Uncertainty Scale-12, and the Fear of Birth Scale. Mediation analysis was conducted using R 4.4.3 software.

**Results:**

A total of 310 surveys were distributed to participants, and ultimately 290 eligible questionnaires were collected, resulting in a response rate of 93.55%. The prevalence of cesarean section preference among participants was 20.69%. The mean score for intolerance of uncertainty was 30.94 ± 7.13, and the mean score for fear of childbirth was 51.50 ± 23.32. This study shows that intolerance of uncertainty and fear of childbirth are both positively associated with a preference for cesarean section. Fear of childbirth mediates the relationship between intolerance of uncertainty and preference for cesarean section.

**Conclusions:**

Intolerance of uncertainty is a positive predictor of preference for cesarean section, and fear of childbirth mediates this association. These findings provide a basis for developing targeted interventions to reduce rates of unnecessary cesarean sections.

## 1 Introduction

In recent decades, the global cesarean section (CS) rate has been rising steadily (1). According to predictive analyses (2), by 2030, 28.5% of women worldwide are projected to give birth via CS. For a long time, China’s CS rate has remained at a high level globally. A survey conducted across 31 provinces in China revealed an overall CS rate of 44.5% (3), far exceeding the WHO recommendation of 10–15% (4).CS plays a crucial role in reducing maternal and neonatal morbidity and mortality in high-risk pregnancies (5). However, as an unnatural and traumatic mode of delivery, it may also entail both short-term and long-term health risks (6). In the short term, mothers may experience complications such as postpartum hemorrhage, infection, and even shock. In the long term, a history of cesarean delivery significantly increases the risk of placenta previa, miscarriage, and stillbirth in subsequent pregnancies (7). Multiple cesarean deliveries further elevate the likelihood of hysterectomy and surgery-related injuries (8). For offspring, existing research indicates that cesarean delivery is a significant risk factor for respiratory infections, asthma, and obesity during childhood (9). Furthermore, non-essential CS not only have negative impacts on maternal and infant health but may also lead to the waste of medical resources and increased economic burden (10, 11). Reducing non-medically indicated CS remains an urgent public health issue in China. Previous studies have shown that women’s delivery preferences during pregnancy can predict their actual mode of delivery (12). Therefore, timely assessment of pregnant women’s delivery preferences is of great significance for China and other countries with high CS rates.

The process from pregnancy to childbirth is highly complex for women and may be accompanied by a range of emotions, including joy, anxiety, and fear (13). Fear of childbirth (FOC) has become a global public health issue, which can have short-term and long-term impacts on women’s health and well-being (14). The prevalence of FOC varies by country, with a study by Huang et al. (15) reporting a rate of 67.1% in China. A substantial body of evidence indicates that FOC exerts numerous adverse effects on women’s physical and mental health. In the short term, FOC can weaken prenatal maternal–fetal attachment (16) and impair sleep quality (17). During labor, it further compromises the childbirth experience (18), prolongs labor duration (19), and increases the risk of adverse neonatal outcomes (14). In the long term, these negative effects extend into the postpartum period, manifesting as reduced quality of life (20), lower rates of successful breastfeeding (21), and elevated risks of postpartum post-traumatic stress disorder (22) and postpartum depression (18). Notably, FOC is often one of the factors leading pregnant women to request CS. Growing evidence shows that fear of childbirth is a major influence on delivery preferences, and high levels of FOC increase pregnant women’s preference for CS (23, 24).

Intolerance of uncertainty (IU) is defined as a cognitive bias (25) that leads individuals to overestimate the likelihood of adverse events in uncertain situations, thereby triggering fear (26, 27). Pregnancy is often an exciting yet uncertain period (28). For expectant mothers with a lower tolerance for uncertainty, facing the unfamiliar situation of childbirth can easily lead to the perception of delivery as a catastrophic event, thereby triggering intense FOC (29). It is worth noting that some positive outcomes have been achieved in intervention studies targeting FOC and IU. For instance, cognitive-behavioral group therapy has been proven effective in reducing IU levels among pregnant women (30). Similarly, systematic reviews indicate that mindfulness-based childbirth education can significantly alleviate fear of childbirth (31). In addition, previous research has shown a positive correlation between IU and the willingness to seek treatment (32). Pregnant women’s preference for a specific mode of delivery is a health-related behavioral intention (33). However, the specific role of IU in childbirth preferences remains unclear.

The Cognition-Affect-Conation (CAC) theory originates from the field of cognitive psychology and is referred to by scholars as the mental trilogy (34). The CAC model primarily comprises three processes: cognitive appraisal, affective response, and conative intention. This theory posits that cognition serves as the foundation for changing conation, affect acts as its driving force, and conation represents the ultimate outcome (35). In the present study, IU is defined as a cognitive bias, FOC is identified as the key affective factor, and preference for mode of delivery constitutes the final health-related conative behavior. The CAC theoretical model has been widely applied to study the formation of individual attitudes and behavioral intentions, demonstrating strong explanatory power (36). Based on this, this study, grounded in the C-A-C framework, proposes the mediating role of fear of childbirth in the relationship between IU and preference for cesarean section. The study has two main objectives (1): to examine the relationship between IU and preference for CS, and (2) to explore the potential mediating role of FOC in the association between IU and CS preference.

## 2 Materials and methods

### 2.1 Study design and participant

This study used a convenience sampling method to enroll pregnant women in the third trimester who attended the outpatient clinic at Wuxi Maternal and Child Health Care Hospital from July to September 2024 as study subjects. Inclusion criteria: (1) age≥18 years; (2) voluntary participation with written informed consent obtained; (3) gestational age ≥ 28 weeks; (4) singleton pregnancy. Exclusion criteria: (1) severe psychiatric disorders; (2) severe pregnancy complications or comorbidities; (3) presence of indications for cesarean section. According to the statistical Kendall sample estimation method (37), considering a 20% rate of invalid questionnaires, a total of 310 pregnant women were ultimately included as study subjects.

### 2.2 Instrument

#### 2.2.1 Sociodemographic information questionnaire

Self-developed by the researcher after reviewing relevant literature, this questionnaire includes items such as the pregnant women’s age, place of residence, educational level, per capita monthly income, parity, history of infertility, employment status, history of cesarean section, and preference for mode of delivery.

#### 2.2.2 Intolerance of uncertainty

IU was measured using the 12-item Intolerance of Uncertainty Scale (IUS-12). The original Intolerance of Uncertainty Scale (IUS) was developed by Freeston et al. (38) in 1994 and later revised into the short form (IUS-12) by Carleton et al. (39) in 2007. This scale was translated and adapted into the Chinese version by Wu (40) in 2016. The Chinese IUS-12 consists of 12 items across three dimensions: Prospective Avoidance (PA), Inhibitory Avoidance (IA), and Prospective Emotion (PE). All items are rated on a 5-point Likert scale. The total score ranges from 12 to 60, with higher scores indicating higher levels of IU. In the current study, the Cronbach’s α for this scale was 0.86, indicating good internal consistency.

#### 2.2.3 Fear of childbirth

Fear of childbirth was assessed using the Fear of Birth Scale (FOBS) (41), which consists of two VAS. Participants were asked about their feelings regarding the upcoming delivery to evaluate worry and fear levels, requiring them to mark their responses on two 100-mm lines (ranging from 0 to 100). The total FOBS score was calculated as the mean score of the two items. A total score of ≥54 is considered indicative of FOC (42). The Cronbach’s α coefficient in this study was 0.90.

### 2.3 Data collection

The survey was conducted face-to-face with voluntary participation. Prior to the study, participants were informed of its purpose and significance, assured of their right to withdraw at any time, and guaranteed strict privacy protection. Questionnaires were distributed on-site, completed independently by participants, and immediately checked for completeness upon collection. Of the 310 questionnaires distributed, 290 were valid, yielding a valid response rate of 93.55%.

### 2.4 Ethical recognition

This study was approved by the Medical Ethics Committee of Wuxi Maternity and Child Health Care Hospital. (Approval number: 2024-01-0515-07).

### 2.5 Statistical analysis

Continuous variables are presented as mean ± standard error (SE), with group comparisons analyzed by independent samples t-tests. Categorical variables are described as counts and percentages [n (%)], with between-group comparisons assessed using chi-square tests. Since the dependent variable (childbirth preference) is categorical, the mediation analysis was conducted in three steps: First, logistic regression was used to examine the effect of intolerance of uncertainty on preference for cesarean section. Second, linear regression was used to test the effect of intolerance of uncertainty on the mediating variable (fear of childbirth level). Finally, both intolerance of uncertainty and fear of childbirth level were included in a logistic regression model to analyze their effects on preference for cesarean section, thereby assessing the mediating role of fear of childbirth. Mediation analysis was conducted using the RMediation package in R software, which is based on the distribution of the product (43, 44). All analyses were performed with IBM SPSS Statistics 26.0 and R 4.4.3, adopting a two-tailed significance threshold of *P* < 0.05.

## 3 Results

### 3.1 Demographic and obstetric characteristics of the study population

The study included 290 pregnant women aged 18 to 43 years, with a mean age of 29.90 ± 3.99 years. Among the participants, 87.59% of the pregnant women resided in urban areas, 84.14% had an education level of college or above, and 75.52% had a per capita monthly income of ≥5000 CNY. In terms of employment status, 76.21% were employed and 23.79% were unemployed. Furthermore, regarding previous experiences, 68.97% were primiparous, 10.34% had a history of infertility, and 7.24% had a previous cesarean section (**Table 1**).

**Table 1:** Sociodemographic and Obstetrics Characteristics of the Participants (n=290).

Variables	n	%
Age (years)		
>30	115	39.66
≤30	175	60.34
Residence area		
Urban	254	87.59
Rural	36	12.41
Educational level		
Junior high school or below	17	5.86
Secondary specialized or high school education	29	10.00
College/bachelor’s degree	220	75.86
Graduate degree or above	24	8.28
Per capita monthly income (CNY)		
<3000	4	1.38
3000-4999	67	23.10
≥ 5000	219	75.52
Employment		
Yes	221	76.21
No	69	23.79
Parity		
Nulliparous	200	68.97
Multiparous	90	31.03
Infertility history		
Yes	30	10.34
No	260	89.66
Cesarean history		
Yes	21	7.24
No	269	92.76

### 3.2 Mean scores for intolerance of uncertainty and fear of childbirth among pregnant women

The mean scores for fear of childbirth and intolerance of uncertainty among pregnant women were 51.50 ± 23.32 and 30.94 ± 7.13, respectively, as shown in **Table 2**.

**Table 2:** Fear of childbirth and intolerance of uncertainty scores of pregnant women (n = 290).

Variables	Items	Total Score
Fear of childbirth	12	51.50 ± 23.32
Intolerance of uncertainty	2	30.94 ± 7.13

### 3.3 Characteristics of pregnant women by mode of delivery preference

Among the participants, 230 women (79.31%) preferred vaginal birth (VB) while 60 (20.69%) preferred cesarean section (CS). As presented in **Table 3**, the two groups showed no statistically significant differences in age, educational level, per capita monthly income, employment, parity, or residential area.

**Table 3:** Characteristics of pregnant women stratified by preference for mode of delivery.

Variable	VB (N = 230)	CS (N = 60)	*P*-value
Age (years)			0.123
>30	86(37.39%)	29(48.33%)	
≤30	144(62.61%)	31(51.67%)	
Residence area			0.808
Urban	202(87.83%)	52(86.67%)	
Rural	28(12.17%)	8(13.33%)	
Educational level			0.263
Junior high school or below	14(6.09%)	3(5.00%)	
Secondary specialized or high school education	21(9.13%)	8(13.33%)	
College/bachelor’s degree	179(77.83%)	41(68.33%)	
Graduate degree or above	16(6.96%)	8(13.33%)	
Per capita monthly income (CNY)			0.245
<3000	2(0.87%)	2(3.33%)	
3000-4999	55(23.91%)	12(20.00%)	
≥ 5000	173(75.22%)	46(76.67%)	
Employment			0.664
Yes	174(75.65%)	47(78.33%)	
No	56(24.35%)	13(21.67%)	
Parity			0.666
Nulliparous	160(69.57%)	40(66.67%)	
Multiparous	70(30.43%)	20(33.33%)	
Infertility history			<0.001
Yes	15(6.52%)	15(25.00%)	
No	215(93.48%)	45(75.00%)	
Cesarean history			<0.001
Yes	3(1.30%)	18(30.00%)	
No	227(98.70%)	42(70.00%)	
Fear of childbirth	44.35 ± 19.43	78.92 ± 15.30	<0.001
Intolerance of uncertainty	29.16 ± 5.90	37.77 ± 7.32	<0.001

VB, vaginal birth; CS, cesarean section.

### 3.4 Mediation analyses

Conducting a logistic regression analysis with CS preference (Y) as the dependent variable and IU (X) as the independent variable yielded c = 0.217, SE(c) = 0.037. Performing a linear regression analysis with FOC (M) as the dependent variable and IU (X) as the independent variable resulted in a coefficient a = 1.396, SE(a) = 0.176. Finally, conducting a logistic regression analysis with CS preference (Y) as the dependent variable and IU (X) and FOC (M) as the independent variables produced coefficients b = 0.112, c’ = 0.125, SE(b) = 0.020, and SE(c’) = 0.044 (**Table 4**).

**Table 4:** Using the product distribution method to test the mediating effect.

Parameter		Estimate	SE	*P*	95% CI
X-Y (logistic regression)		0.217	0.037	<0.001	1.160-1.344
X-M (linear regression)		1.396	0.176	<0.001	1.050-1.742
X+M-Y (logistic regression)	X-Y	0.125	0.044	0.005	1.043-1.242
	M-Y	0.112	0.020	<0.001	1.081-1.168

X represents intolerance of uncertainty (IU), M represents fear of childbirth, and Y represents cesarean section (CS) preference.

OR, odds ratio; 95% CI, 95% confidence interval; SE, standard error

**Figure 1:**
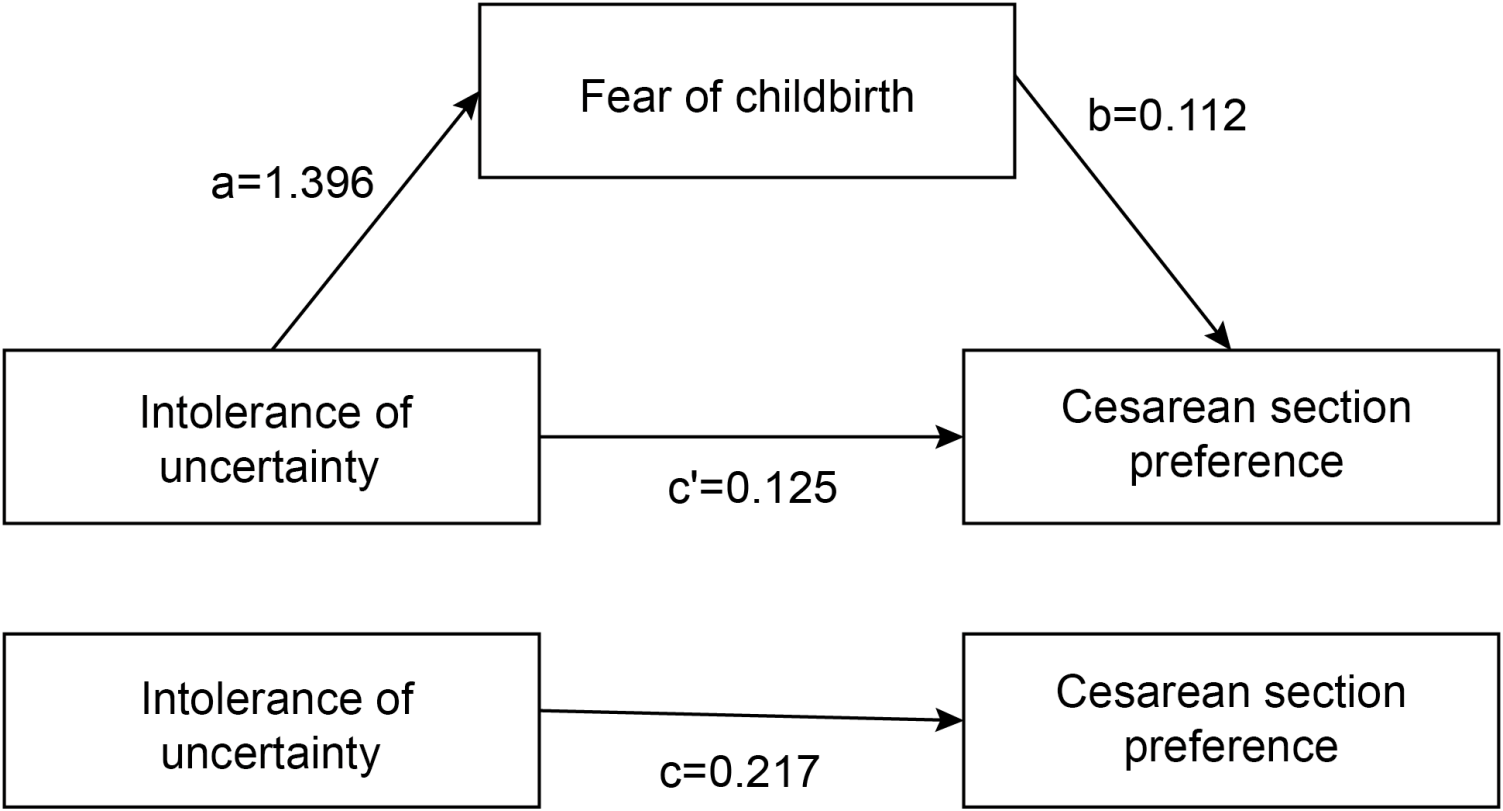
Mediation model diagram of fear of childbirth between pregnant women's intolerance of uncertainty and preference for cesarean section.

The total effect of IU on CS preference (p < 0.001) and the direct effect (p = 0.005) were both statistically significant. Based on the product distribution method of the RMediation package, the 95% confidence interval for the mediating effect is [27.68337, 66.24118], which does not include zero. Therefore, the mediating role of FOC between IU and CS preference was significant. The analysis of the mediating effect pathway is shown in **Figure 1**.

## 4 Discussion

In this study, we explored the relationships among IU, FOC, and CS preference, and examined the mediating role of FOC in the association between IU and CS preference, thereby revealing the potential pathways through which IU influences CS preference.

The present study showed a CS preference rate of 20.69%, which is close to the findings of Sun et al. (33), but lower than those reported by Welay et al. (45) in a study conducted in eastern Ethiopia. This discrepancy may be attributed to regional differences in the sociocultural characteristics of the study populations. Latifnejad et al. (46) argue that social, religious, and cultural beliefs significantly influence pregnant women’s delivery preferences. In this study, the mean IU score was 30.94 ± 7.13, which is higher than the result reported by Flink et al. (47). This may be related to regional differences in the sociocultural context, clinical characteristics, and healthcare environment of the participants. The mean fear of childbirth score among pregnant women in this study was 51.50 ± 23.32, higher than that reported by Zeng et al. (48). This difference may be attributed to variations in the characteristics of the study populations. Pregnant women’s preferences for childbirth can influence the actual mode of delivery. A deeper understanding of these preferences not only helps clinicians better understand pregnant women’s psychological needs and decision-making motivations, but also provides scientific evidence for policymakers, enabling the development of more targeted interventions and health guidance strategies.

In this study, we found that women with higher levels of IU are more likely to prefer CS as their mode of delivery. A previous qualitative study exploring the perceptions of women and healthcare professionals regarding CS delivery (49) indicated that IU can influence pregnant women’s cognitive appraisal of childbirth, thereby increasing their preference for CS. Furthermore, existing evidence suggests that pregnant women with lower tolerance for uncertainty tend to overestimate the likelihood of negative outcomes when facing the unpredictable nature of childbirth. This cognitive bias not only intensifies psychological distress but also undermines their confidence in effectively coping with labor (26), and childbirth self-efficacy has been shown to be negatively associated with CS preference (33). Therefore, enhancing pregnant women’s tolerance for uncertainty may help reduce their preference for CS.

This study shows that FOC is positively associated with preference for CS, indicating that pregnant women with higher levels of FOC tend to have a stronger preference for CS. This finding is consistent with the report by Chen et al. (50). The formation of FOC is a complex psychological process, often involving fear of pain, loss of control, etc. (51). Wigert et al. (13) reported that pregnant women with FOC are more likely to view CS as a controllable, predictable option that avoids the anticipated pain of labor. Furthermore, high levels of FOC are also associated with prenatal anxiety and depression (52, 53), and these psychological conditions themselves may influence childbirth preferences, leading pregnant women to prefer CS as a way to end pregnancy more quickly during the course of their pregnancy (54). Larsson et al. (55) demonstrated that interventions targeting FOC in pregnant women can effectively reduce their preference for CS. Therefore, healthcare professionals should place significant emphasis on prenatal FOC, and through measures such as antenatal health education and psychological counseling, promptly identify women at risk and provide necessary interventions and support. These efforts may help reduce CS preference, minimize unnecessary cesarean deliveries, and ultimately improve maternal and neonatal health outcomes.

According to the CAC Theory, emotions play a crucial role as a bridge connecting cognition and behavioral intention (56). The mediation analysis based on cross-sectional data in this study indicates that fear of childbirth may potentially serve as a mediator between intolerance of uncertainty and the preference for cesarean section. Specifically, the data reveal that IU is not only directly associated with a stronger preference for CS but is also indirectly linked to it through the exacerbation of FOC. This pattern suggests that for pregnant women with high levels of IU, the inherent uncertainties of the childbirth process may be more likely to be appraised as threatening, thereby intensifying their FOC (29), which in turn is associated with an increased inclination to choose cesarean section. This finding provides a plausible explanatory framework for understanding the psychological mechanisms underlying the preference for CS without medical indications, while also pointing to potential directions for clinical intervention. Therefore, in prenatal care, it is recommended to particularly focus on pregnant women with high IU traits, providing them with targeted psychological support and systematically implementing screening and management for FOC. Interventions based on the findings of this study could be explored as a potential pathway to reduce the rate of CS without medical indications.

Our study has some limitations. First, this study employed a convenience sampling method, with all participants recruited from the same tertiary hospital. Although this approach enhances data collection efficiency, it may introduce selection bias, thereby limiting the generalizability of the findings. Consequently, the conclusions of this study should be interpreted with caution when extended to broader populations. Future research should further validate the generalizability of the results through multi-center, random sampling designs and by including pregnant women with diverse characteristics. Second, as a cross-sectional study, this research can only reveal statistical associations and theoretical mediating pathways between variables, without establishing causal relationships. Subsequent studies should adopt longitudinal tracking or experimental designs to further validate the underlying mechanisms among the variables. Additionally, this study did not control for other potential confounding factors that may simultaneously influence IU and FOC, such as clinical anxiety, depressive symptoms, or personality traits. Future research should incorporate more comprehensive psychological assessment tools and control for such variables in statistical models to more accurately evaluate the independent effects of IU on FOC and CS preferences.

## 5 Conclusions

This study indicates that pregnant women’s IU and FOC are positively associated with preference for CS.FOC mediates the relationship between IU and CS preference. These findings provide a basis for developing targeted interventions to reduce rates of unnecessary cesarean sections.

## Data Availability

The raw data supporting the conclusions of this article will be made available by the authors, without undue reservation.
